# Tetrandrine citrate suppresses lung adenocarcinoma growth via SLC7A11/GPX4-mediated ferroptosis

**DOI:** 10.1007/s12672-023-00691-6

**Published:** 2023-06-02

**Authors:** Xiaocong Mo, Di Hu, Kaisheng Yuan, Juyu Luo, Cheng Huang, Meng Xu

**Affiliations:** 1 Department of Oncology, the First Affiliated Hospital of Jinan University, Jinan University, Guangzhou, 510000 Guangdong China; 2 Department of Neurology and Stroke Centre, the First Affiliated Hospital of Jinan University, Jinan University, Guangzhou, 510000 Guangdong China; 3 Department of Metabolic and Bariatric Surgery, the First Affiliated Hospital of Jinan University, Jinan University, Guangzhou, 510000 Guangdong China

**Keywords:** Lung adenocarcinoma, Ferroptosis, Tetrandrine citrate, SLC7A11/GPX4

## Abstract

Ferroptosis is a mode of programmed cell death that plays a crucial role in tumor biology processes. Although tetrandrine citrate (TetC) has been demonstrated to exert anti-tumor effects, it is still unclear whether TetC inhibits lung adenocarcinoma (LUAD) progression by inducing ferroptosis. The study showcased the inhibitory effect of TetC on the viability and progression of tumor cells, including intracellular iron overload, accumulation of reactive oxygen species (ROS), over-expression of malondial-dehyde (MDA), and depletion of glutathione (GSH). Notably, TetC-induced cell death was clearly reversed by three different ferroptosis-related inhibitors. TetC also induced changes in the mitochondrial morphology of LUAD cells, similar to those observed in typical ferroptosis. Further analysis through Western blot (WB) and Immunofluorescence (IF) assays identified that TetC inhibited the expression and fluorescence intensity of both solute carrier family 7 (SLC7A11) and glutathione peroxidase-4 (GPX4). More importantly, over-expression of SLC7A11 could rescue the TetC-induced ferroptosis. Finally, in our vivo experiment, we discovered that TetC significantly slowed the growth rate of subcutaneous transplanted A549 cells, ultimately proving to be biosafe. In conclusion, our study first identified the mechanism by which TetC-induced ferroptosis in LUAD via SLC7A11/GPX4 signaling.

## Highlights

- Ferroptosis as an important method contributed to tetrandrine citrate-induced LUAD cell death.
- Tetrandrine citrate induced ferroptosis in LUAD cells via SLC7A11/GPX4 axis.
- Tetrandrine citrate has therapeutic effect on LUAD and, and exhibits a high degree of biosafety.

## Introduction

Lung adenocarcinoma (LUAD) is the most common subtype of lung cancer, which is responsible for the majority of cancer-related deaths worldwide [1, 2]. Despite the emergence of many new diagnostic tools for LUAD in recent years, the prognosis of patients remains uncertain. Therefore, it is crucial to develop new therapeutic targets and effective drugs to improve the prognosis of LUAD.

Ferroptosis is a non-apoptotic programmed cell death that is dependent on iron. It differs from other types of cell death, such as pyroptosis, apoptosis, necroptosis, and autophagy [3]. This special form of cell death is obviously characterized by aberrant iron metabolism, depletion of the glutathione (GSH), abnormal lipid peroxidation (LP) and altered mitochondrial morphology [4, 5]. Additionally, recent studies have demonstrated that ferroptosis can be directly triggered by abnormal accumulation of LP through inhibition of glutathione peroxidase-4 (GPX4) [6]. The system X_c_^−^, consists of two subunits (SLC7A11, light chain; SLC3A2, heavy chain), antiport cystine/glutamate in the synthesis of GSH [7]. SLC7A11 mainly transports cystine, which is reduced to cysteine to synthesize glutathione. And SLC3A2 maintains the stability of SLC7A11 protein. GSH is an important non-enzymatic antioxidant. In addition to scavenging free radicals, GSH is a cofactor in the GPX4-mediated reduction of lipid hydroperoxides (LOOH). Excessive accumulation of LOOH on the cell membrane can lead to ferroptosis. By catalyzing the oxidation of GSH to GSSG, GPX4 reduces lipid hydroperoxides to lipid alcohols to neutralize oxidized substances in cells, thereby inhibiting ferroptosis. Inactivation of SLC7A11 induces ferroptosis in a variety of cancer cells. In contrast, overexpression of SLC7A11 promoted GSH biosynthesis and ferroptosis resistance in cancer cells [8, 9]. SLC7A11/GPX4 are two of the most important antioxidant enzymes. Inhibition of SLC7A11/GPX4 triggers ferroptosis, whereas activation of SLC7A11/GPX4 inhibits ferroptosis. Abundant evidences have suggested that ferroptosis also exerts potential anti-cancer effect [10]. For example, in osteosarcoma, SLC7A11 expression is found to be upregulated, leading to increased cystine uptake and glutathione production, which provides protection against oxidative stress in tumor cells. GPX4 acts as a downstream of SLC7A11, and its expression is also increased, which contributes to cancer resistance to chemotherapy-induced ferroptosis [11]. Similarly, in breast cancer, expression of SLC7A11 and GPX4 were elevated, which were found to play a critical role in promoting cancer cell survival and resistance to ferroptosis [12]. Therefore, down-regulation of SLC7A11/GPX4 to initiate ferroptosis may become a new breakthrough for the treatment of LUAD.

Tetrandrine (Tet) is a low-toxicity Chinese clinical isolated from the plant *Stephania tetrandra S. Moore*, which has been extensively used for treating autoimmune disorders and lung fibrosis [13]. In recent years, Tet has been identified to have certain anti-tumor activities. However, its application in the actual clinical field is limited due to its hydrophobic character [14, 15]. Tetrandrine citrate (TetC) is a highly water-soluble compound that has demonstrated effective anti-tumor activity in human glioma [16]. Chen et al. have detected that Tet could suppress A549 cell viability and induce apoptosis in lung cancer [17, 18]. Yin et al. have evidenced that TetC triggered ferroptosis cell death in breast cancer cells [19]. However, it is still unclear whether TetC inhibits LUAD progression via inducing the underlying mechanism of ferroptosis.

In our current work, we examined the role of TetC in the LUAD progression as well as the effect of TetC on ferroptosis in LUAD. Our findings revealed that TetC increased the level of ROS and MDA and triggered ferroptosis through inhibiting SLC7A11 to down-regulate GPX4. These results provide novel pharmaceutical targets for the comprehensive treatment of LUAD.

## Materials and methods

### Cell culture and transfection

LUAD cell lines (A549 and H1299) were generously provided by Dr. Feng Ma. The two LUAD cells were then cultured in RPMI-1640 medium (Hyclone; GE Healthcare) containing 10% FBS and maintained in 37 ℃ with 5% CO_2_.These cells were digested with pancreatin (Life-iLab, Shanghai, China). Oe-SLC7A11 and its oe-ctrl, si-SLC7A11 and its si-ctrl were synthesized by GenePharma (Shanghai, China). Subsequent over-expression and siRNA transfections were then carried out following comprehensive reference instructions.

### Cell proliferation assay

To evaluate cell viability, A549 and H1299 cells were seeded in 96-well plates and treated with about seven doses of TetC (0–30 µM) for 12, 24, 48 h. Additionally, cells were treated with Fer-1 (5 µM), DFO (20 µM) and Lip (2 µM) for 24 h to assess cell proliferation. After treated with 10 µL of CCK-8 reagent for 2 h, we read the absorbance at 450 nm using CYTATION 5 Reader (BioTek, USA).

### Colony formation assays

LUAD cells (A549 and H1299) were seeded onto 6 cm dishes, next day, treated with three different concentrations of TetC (0, 5, 10 µM) for about 2 weeks. After being washed with phosphate-buffered saline (PBS) 3 times for about 5 min each, 4% paraformaldehyde (10 min) and crystal violet solution (15 min) were applied to fix and stain the two cells respectively. Finally, the mobile phone took photos to save the data for subsequent analysis.

### Measurement of ROS

Cellular ROS levels of the two treated LUAD cells (0–10 µM TetC; 5 µM Fer-1, 24 h) were measured using ROS assay kit (Beyotime, China). According to the protocol, the treated TetC A549 and H1299 cells were incubated with probe DCFH-DA for 15–20 min and washed repeatedly with serum-free cell medium several times to remove unloaded probe residue. Finally, ROS levels were analyzed with fluorescence microplate reader.

### Detection of malondialdehyde (MDA)

A549 and H1299 cells were treated with three different concentrations of TetC (0, 5, 10 µM) or Fer-1 (5 µM) for 24 h. First, the two cells were lysed and then centrifuged at 10,000 g for 10 min. MDA Assay Kit (S0131, Beyotime) was then approached to measure the specific MDA content in the supernatants of these two cell lines. Finally, the processed samples were tested at 532 nm with sensitive microplate reader and compared to a common standard curve of MDA.

### Determination of glutathione (GSH) levels

The reduction of GSH level in treated A549 and H1299 cells were measured using GSH kit (Beyotime, S0053, China) following the manufacturer’s reference protocol.

### Immunofluorescence (IF) staining

A549 and H1299 cells were treated with three different concentrations of TetC (0, 5, 10 µM) or Fer-1 (5 µM) for 24 h and then cultivated in 24-well plates. Next, the treated cells were fixed with 4% paraformaldehyde for about 10-15 min. After penetrated with permeabilization solution, the two cells were then blocked with 5% pre-formulated bovine serum albumin (Sigma, Germany) for 40 min. The primary antibody was orderly added and mixed overnight at 4 °C. Next day, the samples were incubated with anti-rabbit /mouse IgG secondary antibody (Rabbit: #4417; 1:600; CST; USA. Mouse: #4408; 1:600; CST; USA) for 60 min, and then stained with DAPI (#4083; CST; USA) for 15 min in the dark. Finally, a high-sensitivity confocal microscope (Nikon A1R/A1) was appointed to observe the changes of fluorescence results.

### Western blot

RIPA lysis buffer (RIPA: PMSF = 100:1) was used to extracted the inner protein of LUAD cells. After measured all densities, the prepared proteins (30 µg/lane) were orderly separated by SDS-PAGE (10% or 12%) and then transferred onto pre-cut PVDF membranes. After blocking in skimmed experimental milk (5%) for 2 h at 37 ℃, the targeted membranes were incubated with preconfigured primary anti-FTH (#4393; 1:600; CST; USA), PTGS2 (#12,282; 1:600; CST; USA), DMTI (#15,083; 1:800; CST; USA), SLC7A11 (ab216876; 1:800; Abcam; USA), GPX4 (ab125066; 1:800; Abcam; USA) and β-actin (#4970; 1:3000; CST; USA) overnight at 4 °C. Next day, these were treated with corresponding secondary antibody. Finally, protein signals on the finished membrane were visualized by enhanced ECL kit (4 A Biotech, China).

### RT‑qPCR analysis

Trizol (Beyotime, China) was elected to extract the total RNA from tumour tissues and LUAD cells. RNA was then reverse-transcribed into complementary DNA (cDNA) benefited by SuperScript VILO cDNA Kit. SYBR Green qPCR Master Mix (Applied Biosystems, USA) was adopted to detect the quantitative PCR from the 2^−ΔΔCt^ method. In this, the all primers were listed in Table 1.

**Table 1:** Primer list

Gene	Primers
SLC7A11	Forward: 5'-TCATTGGAGCAGGAATCTTCA -3' Reverse: 5'- TTCAGCATAAGACAAAGCTCCA -3'
GPX4	Forward: 5'- GAGGCAAGACCGAAGTAAACTAC -3' Reverse: 5'- CCGAACTGGTTACACGGGAA-3'
PTGS2	Forward: 5'- TAAGTGCGATTGTACCCGGAC -3' Reverse: 5'- TTTGTAGCCATAGTCAGCATTGT -3'
FTH	Forward: 5'- CCCCCATTTGTGTGACTTCAT -3' Reverse: 5'- GCCCGAGGCTTAGCTTTCATT -3'
GAPDH	Forward: 5'- ATCACTGCCACCCAGAAGAC -3' Reverse: 5'- ACACATTGGGGGTAGGAACA -3'

### Subcutaneous tumor model

All animal experiments were approved and consented by the Ethical Committee of the Laboratory Animal Center of Jinan University, Guangzhou, China. To establish the subcutaneous tumor mouse model, A549 cells were elected and resuspended in a PBS solution (PBS: matrix = 1:1) and then injected into the armpit of BALB/c nude mice with 2 × 10^7^ cells. Seven  days later, the mice were blindly divided into three groups: the control group (saline), TetC group (100 mg/kg/day), and TetC (100 mg/kg/day) + Fer-1(50 mg/kg/day) group. The size of the tumor and body weight were detectedafter the first drug treatment. About 3 weeks later, the blood of tumor-forming mice blood samples was collected from mice to measure the changes of alanine aminotransferase (ALT), creatinine (CRE), urea nitrogen (BUN) and aspartate aminotransferase (AST) in serum to evaluate the safety of TetC.

### Statistical analysis

Differences were assessed using One-way analysis of variance (ANOVA) and Student’s t-test. All analyses were conducted using SPSS 25.0 software and GraphPad Prism 8.0.1. Experiments were performed independently three times. *P* < 0.05 was considered statistically significant.

## Results

### Tetrandrine Citrate reduced the viability and induced the cell death in LUAD cells

**Fig. 1:**
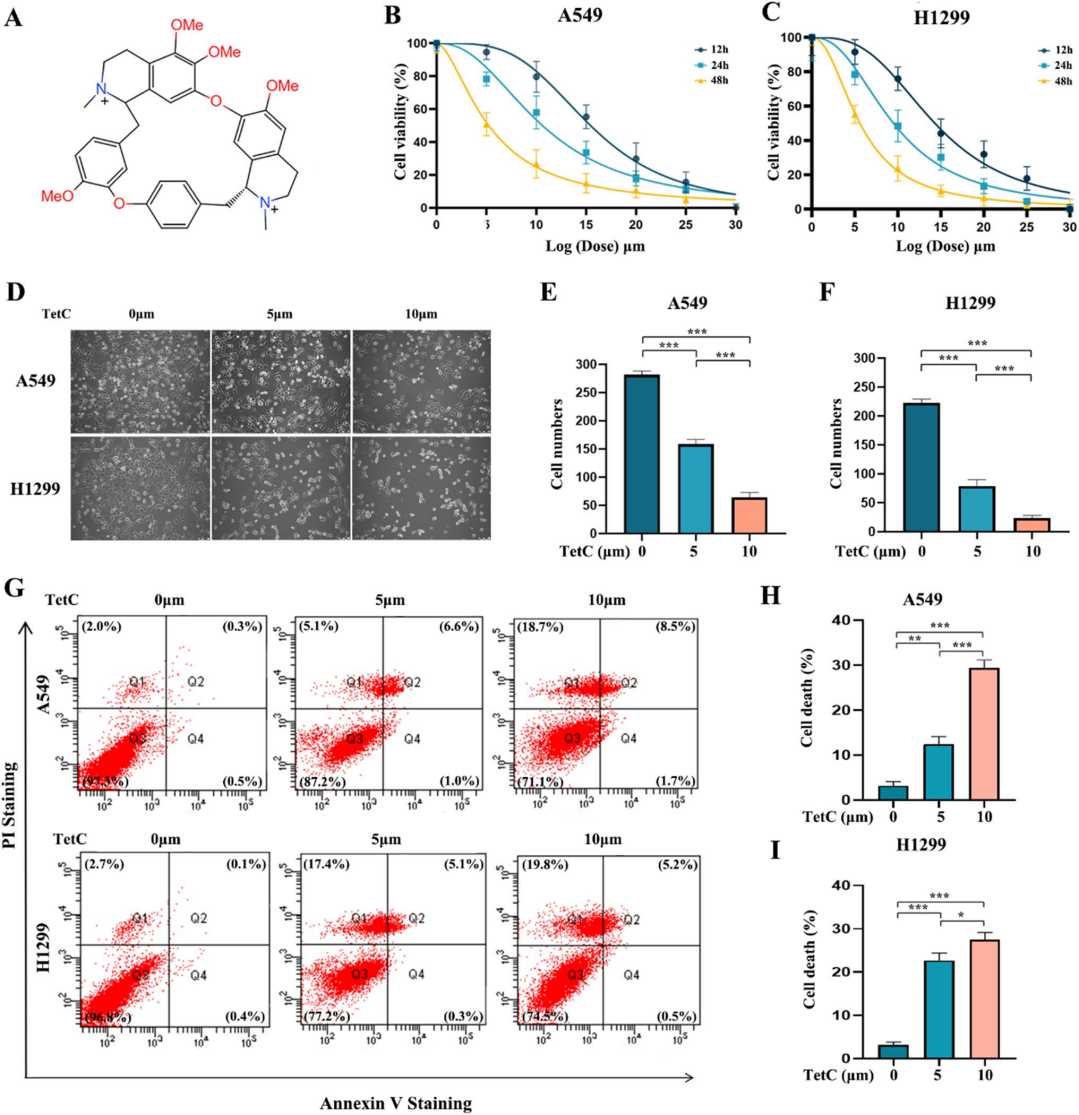
TetC reduced the viability and induced the cell death in LUAD cells. **A** Chemical structure of Tet. **B** CCK-8 assay depicted TetC inhibited A549 cell in a concentration- (0–30 µM) and time- (12 h, 24 h, and 48 h) dependent manner. **C** CCK8 assay depicted TetC inhibited H1299 cell in a concentration- (0–30 µM) and time- (12 h, 24 h, and 48 h) dependent manner. **D–F** Morphologic features of A549 and H1299 cells on microscopy. Cells became obviously round and showed shrunk after TetC (5 µM and 10 µM) treatment for 24 h. **G** A549 and H1299 cells were subjected to TetC (5 µM and 10 µM) for 24 h and followed by Annexin V-FITC/PI assay. **H**,** I** Quantification of Annexin V-FITC/PI in A549 and H1299 cells. ^*^p < 0.05; ^**^p < 0.01; ^***^p < 0.001

Figure 1 A described the internal chemical molecular structure of Tetrandrine. As Tetrandrine is a hydrophobic alkaloid with low solubility in water, Tetrandrine Citrate was formed by mixing Tetrandrine free base with citric acid at 4:1 ratio in ddH_2_O. TetC has a solubility of up to 500 mg/ml in water. Next, in order to investigate the anticancer properties of TetC on LUAD, different doses of TetC were treated in the LUAD cells (A549 and H1299). We found that TetC had the potentiality to restrain the viability of LUAD cells in a concentration-dependent manner (Fig. 1B, C). And the half-maximal inhibitory concentrations (IC50s) in A549 and H1299 cells were 10.610 µM and 9.492 µM, respectively, therefore, 10 µM of TetC concentration were finally selected for the following experiments. In addition, as shown in Fig. 1D–F, TetC obviously prevented the growth of A549 and H1299 cells in a concentration-dependent manner. Furthermore, we utilized the Annexin V-FITC/PI assay to evaluate the effect of TetC on the LUAD cell death. As expected, treatment with TetC increased the amounts of A549 and H1299 cells death compared with the ctrl-group (Fig. 1G–I). Collectively, these data suggested that TetC inhibited the viability and induced cell death in LUAD cells.

### Tetrandrine citrate induced DNA damage and inhibited the clonogenesis of LUAD cells

**Fig. 2:**
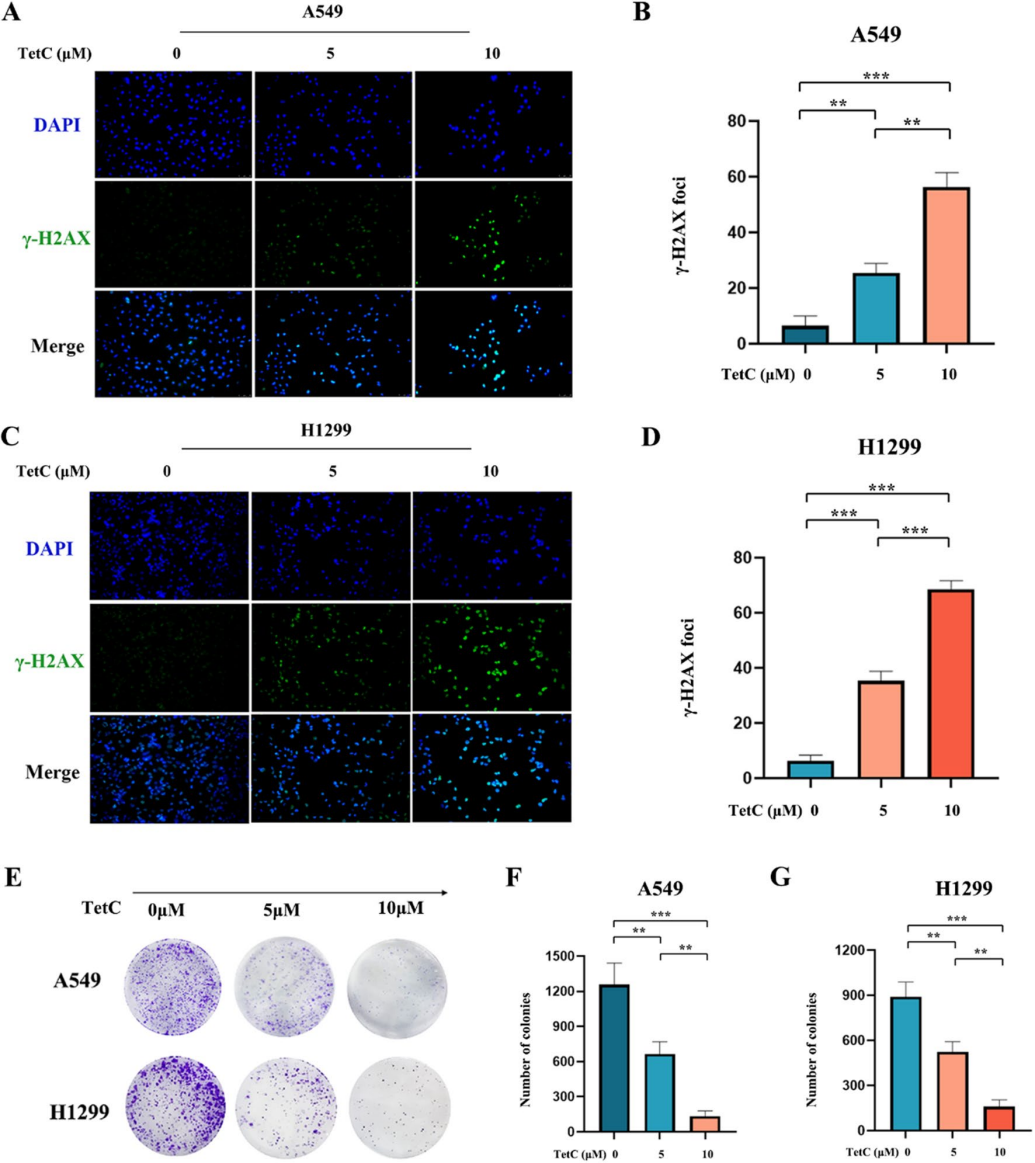
TetC induced DNA damage and inhibited the clonogenesis of LUAD cells. **A** A549 cell were treated with TetC (5 µM and 10 µM) for 24 h and stained with γ-H2AX antibody. DAPI stands for nucleus staining. Scale bar: 10 μm. **B** Quantification of γ-H2AX immunofluorescence in A549 cell. **C** H1299 cell were treated with TetC (5 µM and 10 µM) for 24 h and stained with γ-H2AX antibody. DAPI stands for nucleus staining. Scale bar: 10 μm. **D** Quantification of γ-H2AX immunofluorescence in H1299 cell. **E** The colony formation assay of A549 and H1299 cells were performed under treatment of TetC (5 µM and 10 µM) for 14 days. **F**, **G** Quantification of colony formation in A549 and H1299 cells. ^*^p < 0.05; ^**^p < 0.01; ^***^p < 0.001

Immunofluorescence was used to measure the change of γ-H2AX expression which is a marker for early DNA damage. Results showed that TetC dramatically increased amounts of γ-H2AX foci in a dose-dependent manner of A549 and H1299 cells (Fig. 2A–D). Next, the clonogenic assay was employed to identify the effect of TetC on cell clone. The results depicted in Fig. 2E–G showed that compared with the control group, TetC remarkably inhibited the colony formation of LUAD cells in a dose-dependent manner. In summary, our research has shown that TetC plays a significant role in inducing DNA damage and suppressing the clonogenesis of LUAD cells, highlighting its potential as a valuable tool in cancer treatment and prevention.

### Ferroptosis as an important method contributed to tetrandrine citrate-induced LUAD cell death

**Fig. 3:**
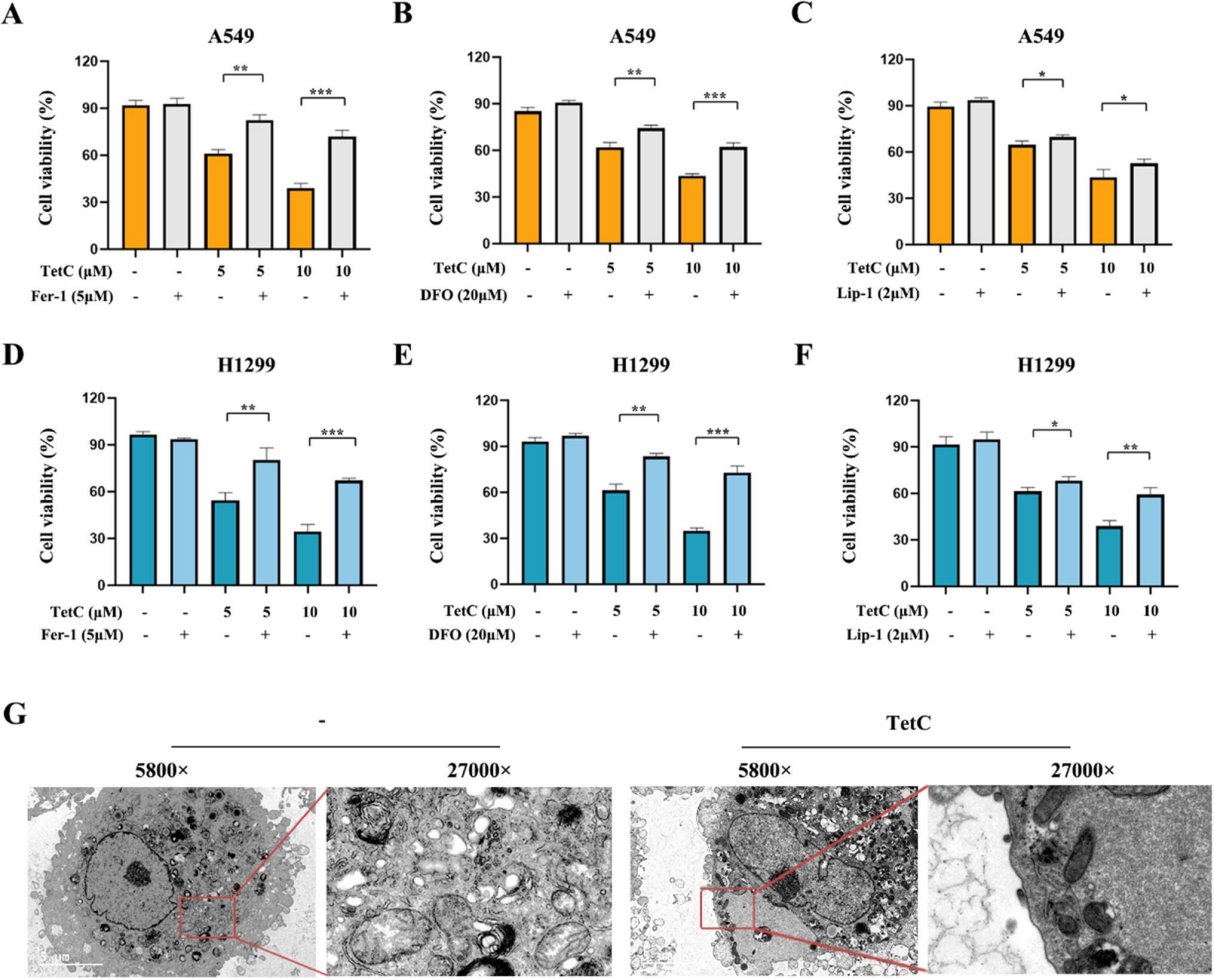
TetC induced ferroptosis in LUAD cells. **A**,** D** The viability of A549 and H1299 cells was detected following TetC (5 µM and 10 µM) with or without Fer-1 (5 µM) treatment for 24 h. **B**,** E** The viability of A549 and H1299 cells was detected following TetC (5 µM and 10 µM) with or without DFO (20 µM) treatment for 24 h. **C**,** F** The viability of A549 and H1299 cells was detected following TetC (5 µM and 10 µM) with or without Lip-1 (2 µM) treatment for 24 h. ^*^p < 0.05; ^**^p < 0.01; ^***^p < 0.001. **G** The ultrastructure of control and TetC (10 µM) treated LUAD cell was observed using TEM. TEM: transmission electronic microscopy

Several studies conducted in the past year have demonstrated that stimulation of ferroptosis plays an irreplaceable function in the chemoresistance of various human cancers [20, 21]. In this work, we observed that TetC-induced LUAD cell death was remarkably blocked by three different ferroptosis-related inhibitors, ferrostatin-1 (Fer-1, 5 μm, ferroptosis inhibitor), liproxstatin-1 (Lip-1, 2 μm, ferroptosis inhibitor) and defer-oxamine (DFO, 20 μm, iron chelator), proving that ferroptosis might be essential for TetC-induced cell death in LUAD (Fig. 3A–F). We then looked into the morphology of the TetC treated cells. As shown the results of transmission electron microscopy (TEM) in Fig. 3G, the treated cells played shrinking mitochondria, condensed mitochondrial membrane densities and diminished or disappeared mitochondria cristae, which are emblematic morphological features of ferroptosis.

**Fig. 4:**
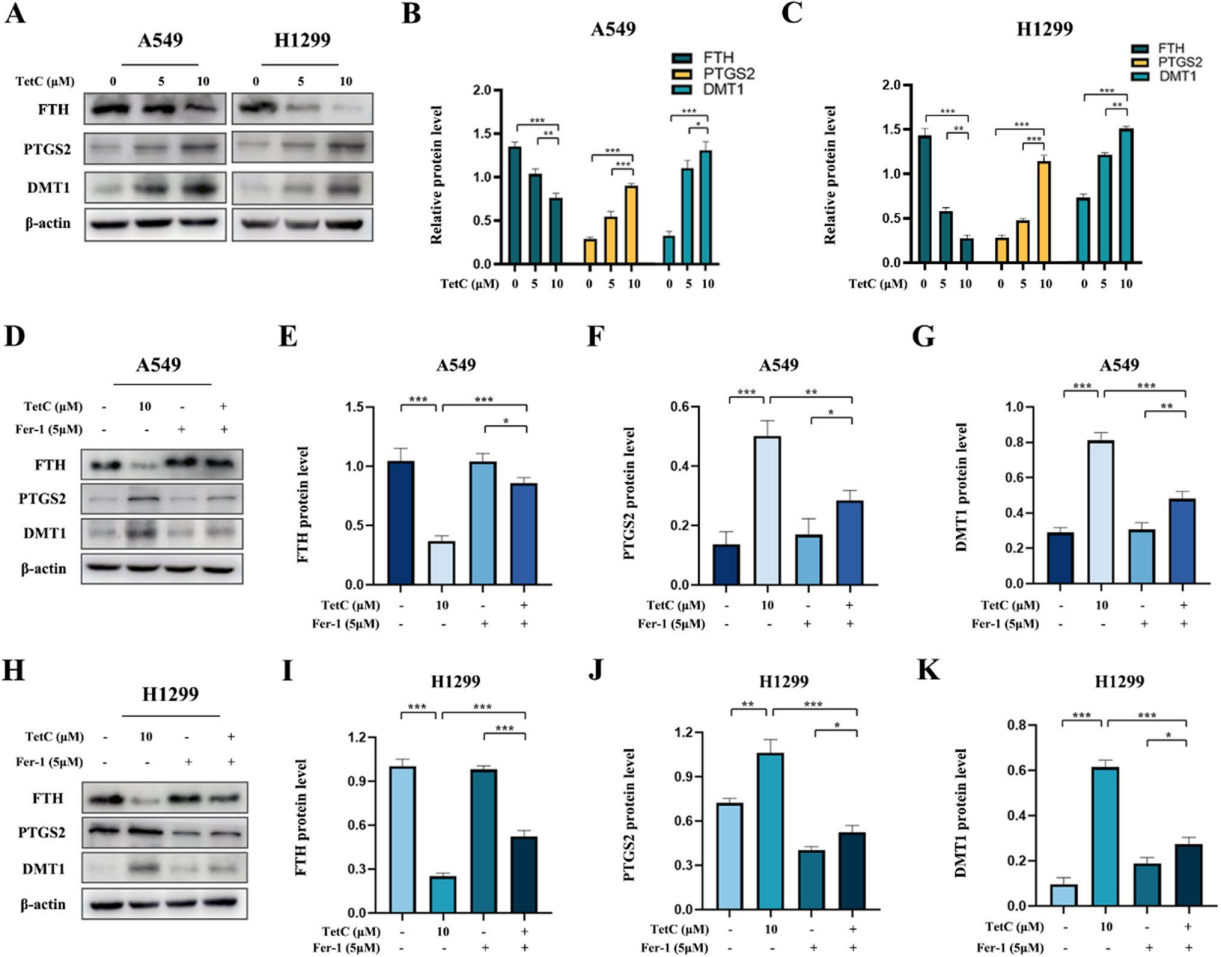
Ferroptosis was triggered by TetC in LUAD cells. **A** The expression of ferroptosis-related proteins in A549 and H1299 cells were detected after TetC (5 µM and 10 µM) treatment for 24 h by western blotting. **B** Quantification of western blotting in A549 cell. **C** Quantification of western blotting in H1299 cell. **D** The expression of ferroptosis-related proteins in A549 cell were detected after TetC (10 µM) treatment with or without Fer-1 (5 µM) for 24 h by western blotting. **E–G** Quantification of western blotting in A549 cell. **H** The expression of ferroptosis-related proteins in H1299 cell were detected after TetC (10 µM) treatment with or without Fer-1 (5 µM) for 24 h by western blotting. **I–K** Quantification of western blotting in H1299 cell. ^*^p < 0.05; ^**^p < 0.01; ^***^p < 0.001

To investigate whether TetC induced LUAD cell death through ferroptosis, we examined ferroptosis-related marker proteins (Ferritin heavy chain, FTH; Prostaglandin endoperoxide synthase 2, PTGS2; Divalent metal transporter 1, DMT1) by western blot. Our data reveled that LUAD cells treated with TetC displayed a dose-dependent increase in the expression of PTGS2 and DMTI, but decrease the levels of FTH (Fig. 4A–C). Furthermore, the addition of Fer-1 could reverse the expression of these proteins induced by TetC treatment (Fig. 4D**–**K).

**Fig. 5:**
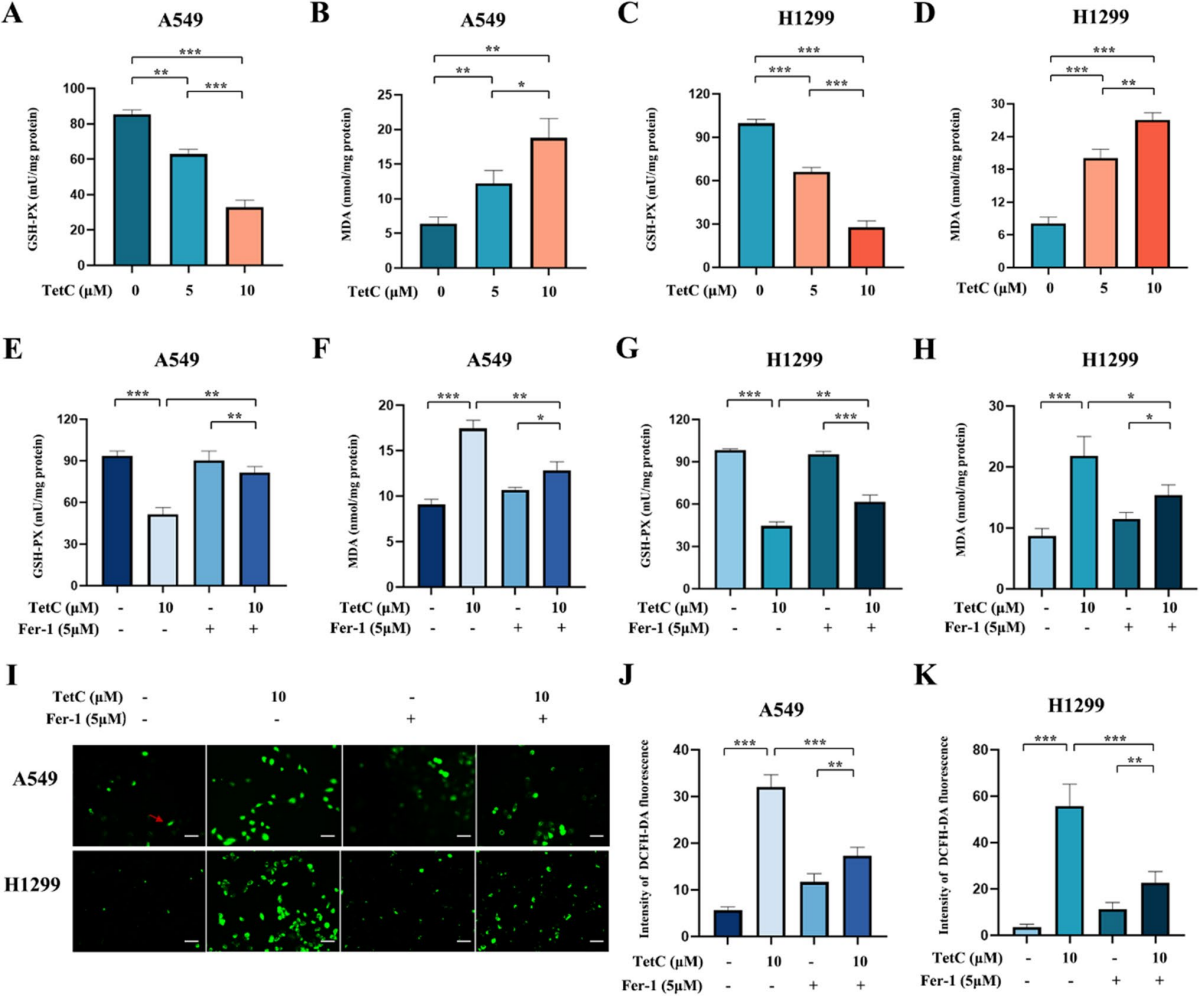
Ferroptosis contributed to TetC-induced cell death in LUAD. **A**,** B** GSH-PX and MDA levels in A549 cell were detected after TetC (5 µM and 10 µM) treatment for 24 h. **C**,** D** GSH-PX and MDA levels in H1299 cell were detected after TetC (5 µM and 10 µM) treatment for 24 h. **E**,** F** GSH-PX and MDA levels in A549 cell were detected after TetC (10 µM) treatment with or without Fer-1 (5 µM) for 24 h. **G**,** H** GSH-PX and MDA levels in H1299 cell were detected after TetC (10 µM) treatment with or without Fer-1 (5 µM) for 24 h. **I–K** ROS level in A549 and H1299 cells was detected after TetC (10 µM) treatment with or without Fer-1 (5 µM) for 24 h with a ROS Assay Kit. ^*^p < 0.05; ^**^p < 0.01; ^***^p < 0.001

As part of the ferroptosis process, it is essential to note the depletion of GSH, as well as the accumulation of MDA and ROS [22]. Thus, the level of GSH, MDA and ROS were measured and observed. As expected, the gradually decreased GSH level and the increased MDA level were observed as TetC concentration (5 and 10 μm) over 24 h (Fig. 5A–D). Moreover, Fer-1 could eliminate these results induced by TetC (Fig. 5E–H). As anticipated, the TetC treatment (10 µM, 24 h) upregulated the levels of ROS, and Fer-1 could eliminate this phenomenon in LUAD cells (Fig. 5I–K). In short, these results reveled that ferroptosis acted as the critical mechanism by which TetC triggered cell death in LUAD.

### Tetrandrine citrate induced ferroptosis in LUAD cells via SLC7A11/GPX4 axis

**Fig. 6:**
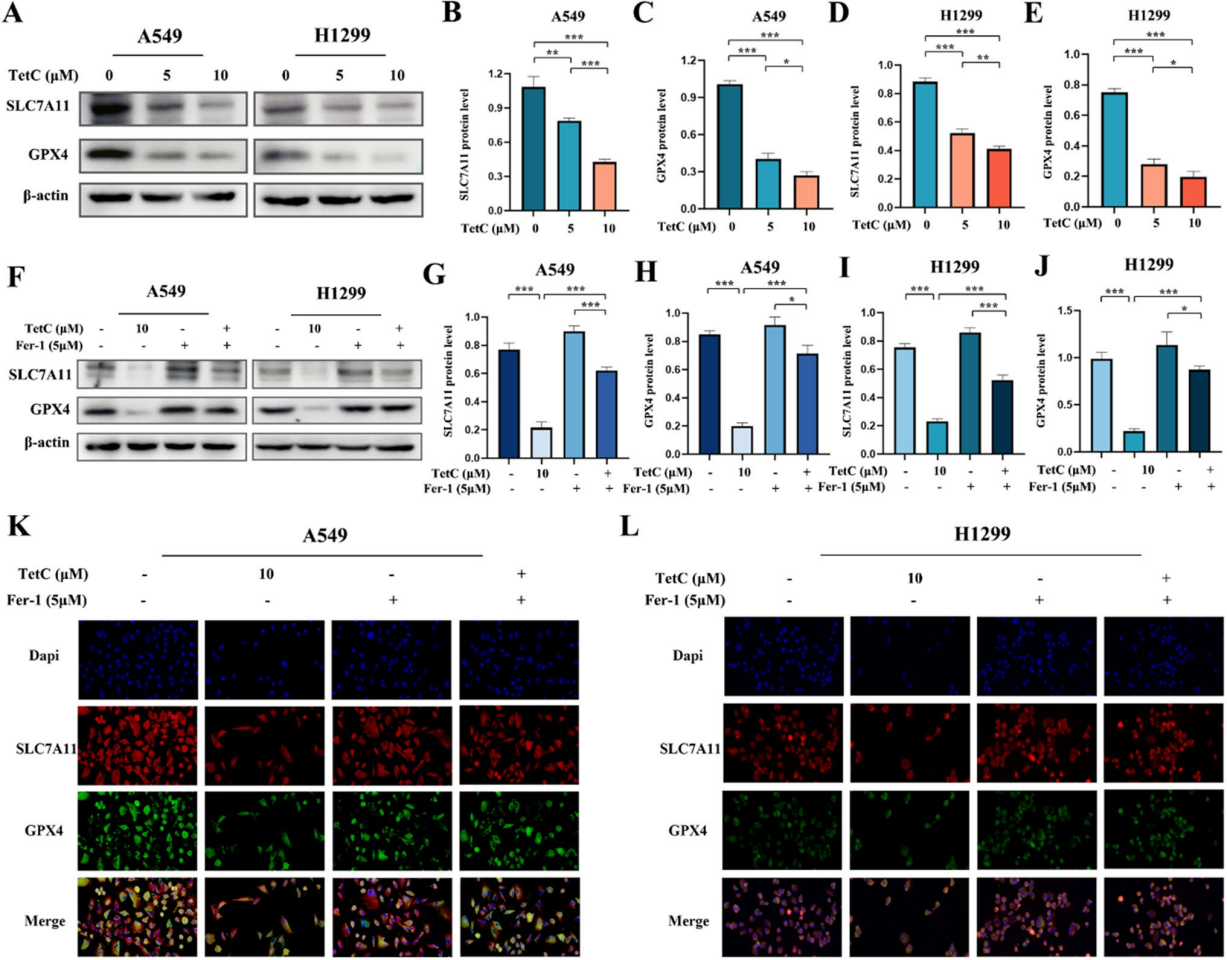
The expression of SLC7A11 and GPX4 in LUAD cells after TetC treatment. **A** SLC7A11 and GPX4 levels in A549 and H1299 cells were detected after TetC (5 µM and 10 µM) treatment for 24 h by western blotting. **B–E** Quantification of western blotting in A549 and H1299 cells. **F** SLC7A11 and GPX4 levels in A549 and H1299 cells were detected after TetC (10 µM) treatment with or without Fer-1 (5 µM) by western blotting. **G–J** Quantification of western blotting in A549 and H1299 cells. **K** The fluorescence intensity of A549 cell was detected after TetC (10 µM) treatment with or without Fer-1 (5 µM) by Immunofluorescence staining. **L** The fluorescence intensity of H1299 cell was detected after TetC (10 µM) treatment with or without Fer-1 (5 µM) by Immunofluorescence staining. ^*^p < 0.05; ^**^p < 0.01; ^***^p < 0.001

**Fig. 7:**
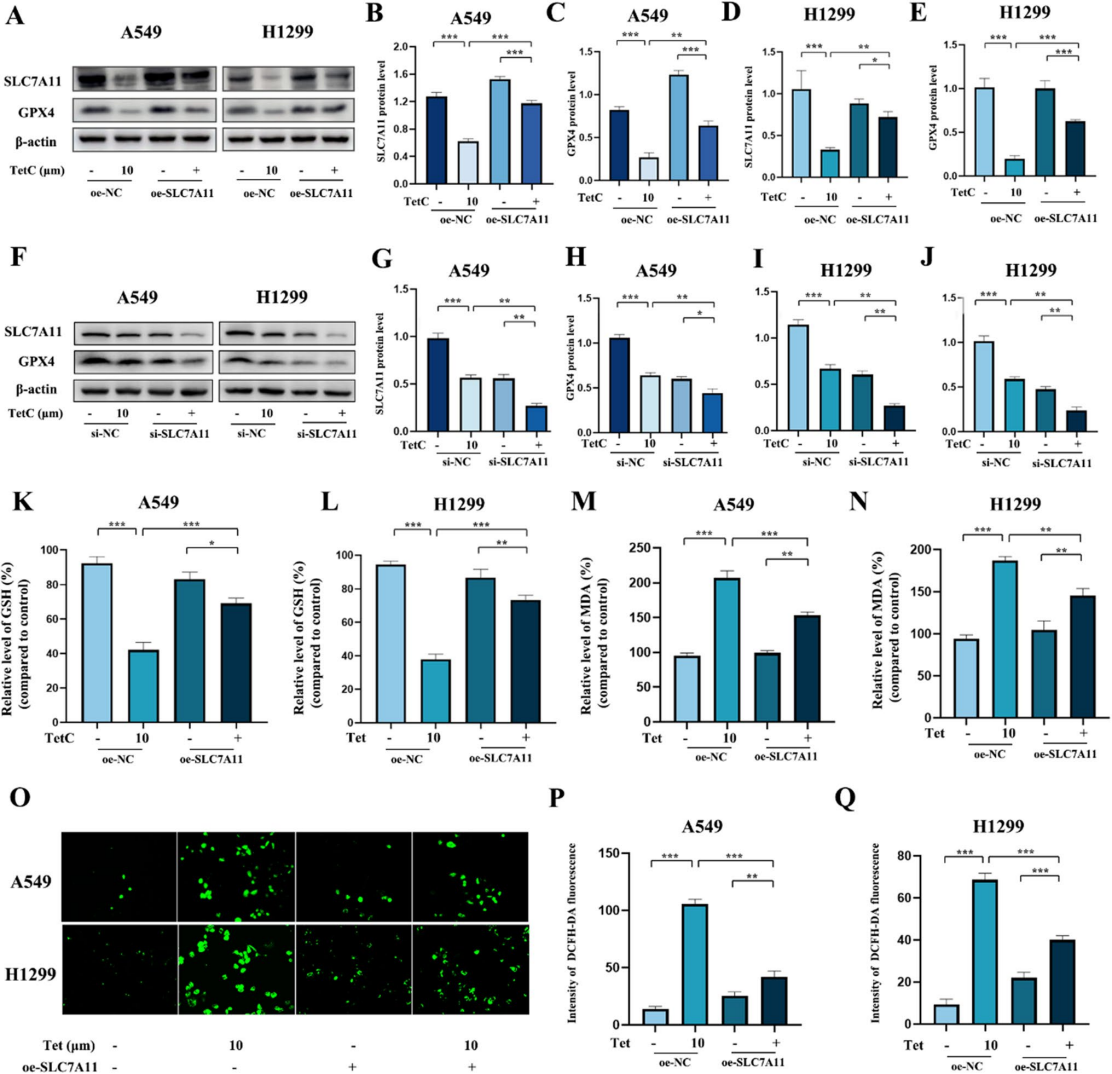
TetC-induced ferroptosis depends on the SLC7A11/GPX4 signaling pathway. **A** SLC7A11 and GPX4 levels in A549 and H1299 cells were detected after TetC (10 µM) treatment with or without oe-SLC7A11 by western blotting. **B-E ** Quantification of western blotting in A549 and H1299 cells. **F** SLC7A11 and GPX4 levels in A549 and H1299 cells were detected after TetC (10 µM) treatment with or without si-SLC7A11 by western blotting. **G–J** Quantification of western blotting in A549 and H1299 cells. **K**,** L** GSH-PX and MDA levels in A549 cell were detected after TetC (10 µM) treatment with or without oe-SLC7A11. **M**,** N** GSH-PX and MDA levels in H1299 cell were detected after TetC (10 µM) treatment with or without oe-SLC7A11. **O–Q** ROS level was detected after TetC (10 µM) treatment with or without oe-SLC7A11 with ROS Assay Kit. ^*^p < 0.05; ^**^p < 0.01; ^***^p < 0.001

To investigate the potential molecular mechanisms of TetC-induced ferroptosis, we selected two pivotal signals from the ferroptosis complicated pathway, SLC7A11 and GPX4, as the focus of our next research. The expression of SLC7A11 and GPX4 were examined by western blot, reveling that they were remarkably decreased following TetC treatment, and could rescue by the addition of Fer-1 in LUAD cells (Fig. 6A–J). Immunofluorescence assay further found that not only the expression level of SLC7A11 and GPX4 proteins were significantly reduced, but also the fluorescence intensity of these two proteins was weakened with TetC treatment, which could be reversed by Fer-1 (Fig. 6K, L). We further transfected SLC7A11 with overexpressing plasmid and the si-RNA targeting SLC7A11 in LUAD cells before TetC treatment to confirm whether TetC-induced ferroptosis occurred through SLC7A11. As indicated in Fig. 7A–E, K, L, SLC7A11 upregulation clearly increased the decline in GPX4 protein level and GSH content triggered by TetC (10 µM, 24 h). Meanwhile, Fig. 7F–J proved that si-SLC7A11 reduced GPX4 protein levels induced by TetC in A549 and H1299 cells. In addition, the overexpression of SLC7A11 remarkably attenuated the TetC-induced (10 µM, 24 h) accumulation of MDA and ROS (Fig. 7M–Q). Taken together, TetC induced ferroptosis in LUAD through regulating the SLC7A11/GPX4 axis.

### Tetrandrine citrate benefited to treating LUAD and triggered ferropotosis in vivo

**Fig. 8:**
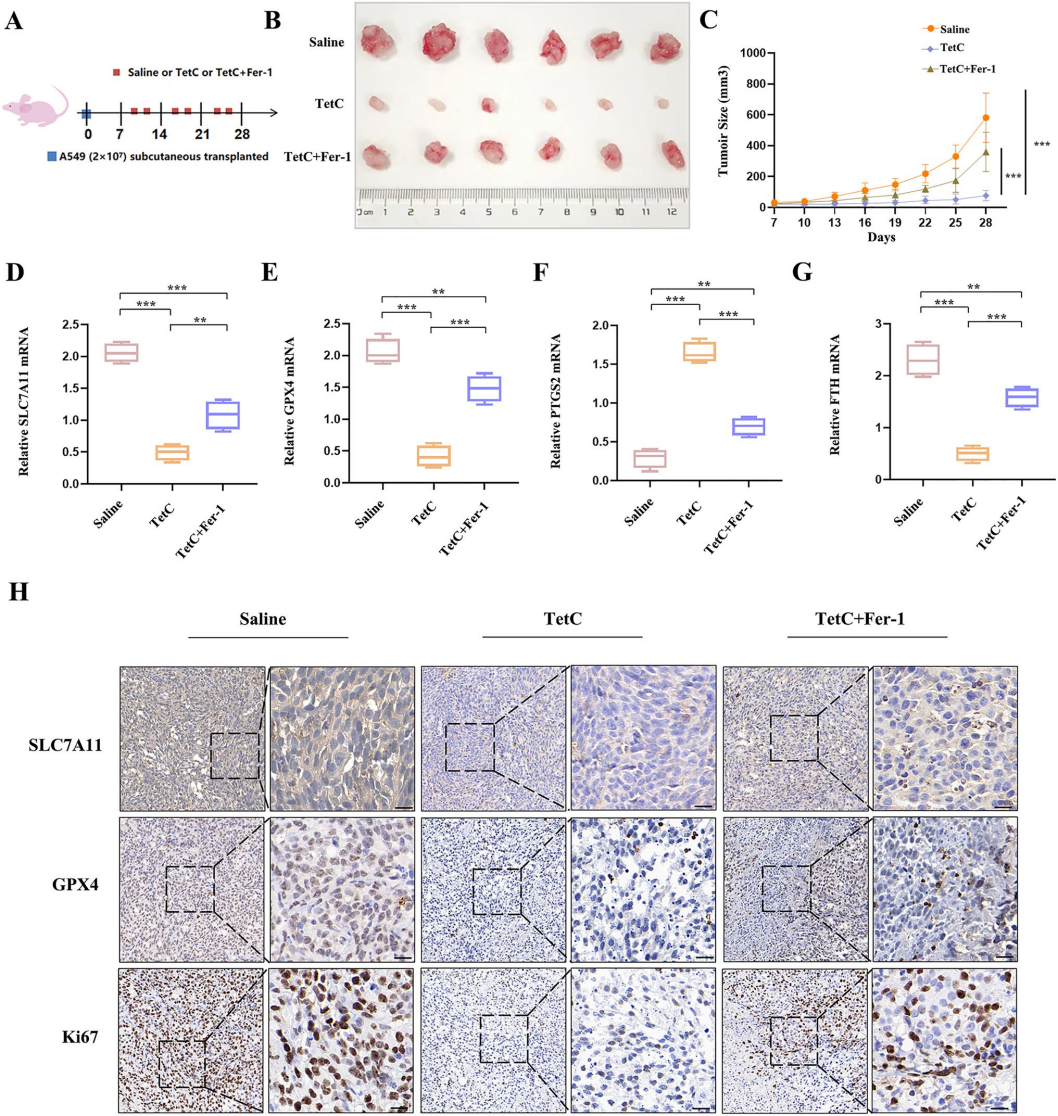
TetC benefited to treating lung cancer in vivo. **A** Once the tumours reached 80–100 mm^3^, the model mice received three different treatments (saline, 100 mg/kg/day TetC, 100 mg/kg/day TetC + 50 mg/kg/day Fer-1) per day until the experiment terminated. **B** The photograph of tumor samples in three different treatment groups. **C** Tumor size was measured. **D–G** RT-qPCR analysis of SLC7A11, GPX4, PTGS2 and FTH mRNA in tumors from three different treatment groups. **H** The SLC7A11, GPX4 and Ki67 were determined by IHC. ^*^p < 0.05; ^**^p < 0.01; ^***^p < 0.001

To evaluate the potential antitumor efficacy of TetC in vivo, the amount of A549 (2 × 10 ^7^) cell was expertly subcutaneously injected into the armpits of experimental BALB/c nude mice (4 weeks age). Once the mice developed 80–100 mm^3^ tumors, they were randomly assigned to receive intraperitoneal injections of TetC (100 mg/kg/day), TetC (100 mg/kg/day) + Fer-1 (50 mg/kg/day) or the same volume of saline for approximately 3 weeks (Fig. 8A). Our data showed that TetC dramatically inhibited tumour growth and Fer-1 could abolished this effect (Fig. 8B, C). Furthermore, we detected the effects of TetC on ferroptosis through RT-qPCR, which also reduced SLC7A11, GPX4 and FTH and increased PTGS2 following the TetC treatment. Moreover, the cell death induced by TetC was apparent eliminated by co-treatment with Fer-1 (Fig. 8D–G). Finally, immunohistochemical (IHC) were performed on tumor sections to further detect protein expression. As demonstrated in the results, The decreased levels of SLC7A11, GPX4 and Ki67 were found following the TetC treatment, which could be reversed by Fer-1 (Fig. 8H). In summary, the above data demonstrated that TetC had the efficacy for inhibiting tumour growth and partly induced ferroptosis in LUAD in vivo.

### The potential biosafety value of tetrandrine citrate on tumour treatment

**Fig. 9:**
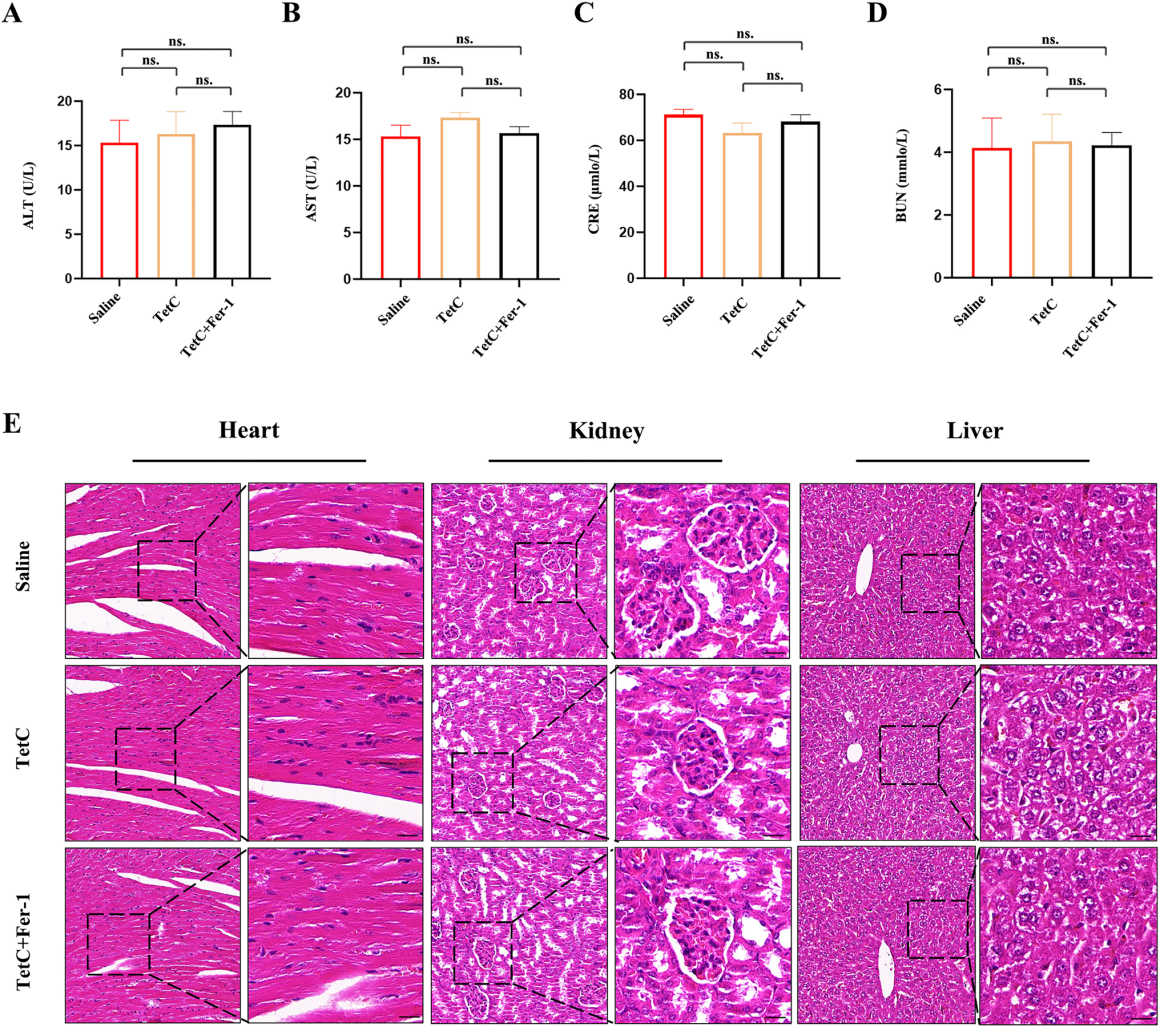
The potential biosafety value of Tetrandrine citrate on tumor treatment. **A**,** B** The levels of ALT and AST were determined from the serum of mice after three different treatment groups (saline, 100 mg/kg/day TetC, 100 mg/kg/day TetC + 50 mg/kg/day Fer-1). **C**,** D** The levels of CRE and BUN were determined from the serum of mice after three different treatment groups (saline, 100 mg/kg/day TetC, 100 mg/kg/day TetC + 50 mg/kg/day Fer-1). **E** HE staining of heart, kidney and liver tissues in mice. ALT and AST: markers of liver function; CRE and BUN: markers of renal function. ^*^p < 0.05; ^**^p < 0.01; ^***^p < 0.001

In further research, we looked forward to exploring whether TetC had a good safety profile for tumor suppression. We collected serum from the TetC-treated mice to examine the function of their liver and kidneys. Our founding indicated that there were no significant changes in serum ALT and AST levels after TetC intervention (Fig. 9A, B). The CRE and BUN results also testified the lack of highlighted renal dysfunction in the three drug-treated mice (Fig. 9C, D). The complete heart, kidney, and liver tissues were dissected and extracted from the mice in each group, and performed HE staining after a series of preliminary treatments. TetC treatment did not trigger significant intrinsic toxic reactions in mice of each group (Fig. 9E). Altogether, our results suggested that TetC had a degree of biosafety in LUAD treatment through the vivo experiment.

## Discussion

In this study, we aimed to investigate the therapeutic effect of TetC on LUAD both in vitro and in vivo, as well as its specific mechanism. Firstly, IC50 of TetC for A549 and H1299 cells were 10.610 µM and 9.492 µM, respectively, indicating that low dose of TetC treatment can lead to massive LUAD cell death. Next, in vitro, we revealed that TetC could induce LUAD cell death, which was remarkably reversed by Fer-1 (5 μm). Moreover, TetC treatment could lead to mitochondrial shrinkage, condensed mitochondrial membrane densities and diminished or disappeared mitochondria cristae in LUAD cells. Consequently, TetC elevated the levels of intracellular and ferroptosis-related factors by increasing DMT1 and PTGS2 expression and decreasing FTH expressions. In vivo perspective, we further showed that TetC significantly delayed the growth rate of subcutaneous transplanted A549 cells and had a degree of biosafety. Finally, TetC was also identified to promote ROS and MDA and reduced GSH accumulation by down-regulating SLC7A11/GPX4 axis.

Ferroptosis is a novel non-apoptotic form of RCD characterized by abnormal iron overload, triggering LP and inhibition of antioxidant enzymes, which might be rescued by antioxidants and its special inhibitors [23]. So far, the detailed molecular mechanism of ferroptosis remains to be investigated. Remarkably, a number of clinical drugs have been identified to trigger ferroptosis in lung cancer by regulating different molecular signaling pathways. For example, Curcumin may induce ROS-dependent ferroptosis via activating the occurrence of autophagy in lung cancer and artemisinin exerted anti-cancer activity through inducing ferroptosis [24, 25]. Moreover, it is known that smaller mitochondria, condensed mitochondrial membrane densities as well as reduced mitochondrial cristae is the signs of ferroptosis morphology [26]. After treatment with TetC, we detected that the morphological changes in treated LUAD cells was similar to that in cells undergoing ferroptosis.

Abnormal iron overload in cells is generally considered to be the origin of ferroptosis. Among them, DMT1 exerted critical function in the transport of ferroptosis [27]. Previous study has shown that DMT1 was a positive regulator of excessive intracellular iron accumulation, causing a burst of ferroptosis [28]. In addition, PTGS2 was detected to be upregulated in ferroptotic cells [29]. Consistent with these previous researches, our findings shown that TetC obviously up-regulated DMT1 and PTGS2 expressions to promote the occurrence of ferroptosis, but Fer-1 could eliminate these results. Furthermore, Ferritin, contained FTH, and its crucial function is to maintain normal iron storage and homeostasis. Tian et al. have demonstrated that over-expression of FTH remarkably inhibited ferroptosis in PC-12 cells [30]. Here, we demonstrated that TetC down-regulated FTH expression in a dose-dependent manner, leading to the disruption of iron stores and homeostasis, thereby triggering ferroptosis. These aforementioned results suggested that LUAD cells undergo ferroptosis with TetC treatment.

Triggering LP, defined as the programmed process by which ROS strongly attack polyunsaturated fatty acids, is the canonical event that drives ferroptosis [31]. Additionally, excessive and abnormal ROS as well as LP end product MDA emerged during the occurrence of ferroptosis [32]. Our findings indicate that TetC is capable of inducing ferroptosis, as evidenced by its ability to increase cellular ROS levels, promote MDA accumulation, and deplete GSH in a dose-dependent manner. However, these aforementioned results would be reversed by the appearance of Fer-1.

**Fig. 10:**
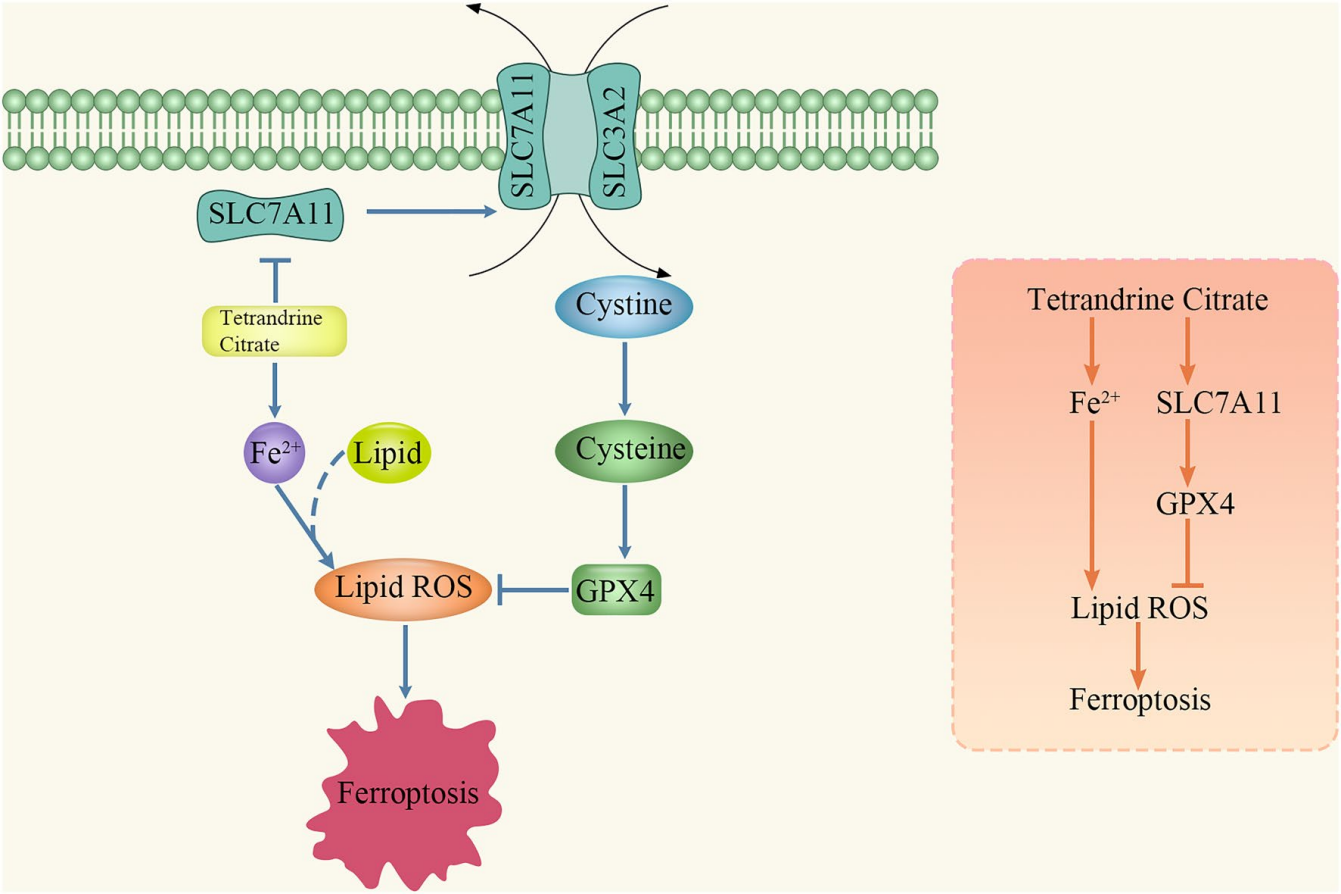
Schematic illustration of TetC-induced ferroptosis via SLC7A11/ GPX4 axis

SLC7A11 plays a crucial role in promoting the synthesis of GSH, which helps counteract oxidative stress and inhibits ferroptosis in various cancer cells [33]. Lung et al. demonstrated that repression of SLC7A11 would trigger tumoral LP and ferroptosis [34]. As an important non-enzymatic antioxidant, GSH not only gets rid of excess free radicals, but also assists the smooth progress of lipid hydroperoxides (LOOH) reduction reactions involving GPX4 [35]. In addition, Li et al. surprisingly found that GPX4 can achieve the goal of preventing ferroptosis by converting lipid hydroperoxides into non-toxic and harmless lipid alcohols through a series of molecular procedures [36]. The research based on the inhibition of ferroptosis by the antioxidant enzyme SLC7A11/GPX4 axis may be a breakthrough in the treatment of lung cancer. Yuan et al. identified that DHA could induce ferroptosis of lung cancer cells by inactivating PRIM2/SLC7A11 axis [37]. Wang et al. also detected that CREB inhibited LUAD ferroptosis via activating GPX4 transcription [38]. Unexpectedly, we found TetC led to ferroptosis in LUAD cells by reducing GPX4 expression, which in turn caused ROS and MDA accumulation, GSH depletion, which were consistent with previous studies. However, over-expression of SLC7A11 obviously rescued LUAD cells from ferroptosis caused by TetC. Therefore, we inferred that TetC triggered LUAD cell ferroptosis might be regulate the SLC7A11/GPX4 axis (Fig. 10).

The results of this study provide a new direction for the development of cancer therapy targeting the SLC7A11/GPX4 pathway. At the same time, future studies can explore the development of new therapeutic methods targeting this pathway, the relationship between this pathway and platinum resistance, and the possibility of combining TetC with other anticancer drugs. However, no clinical study has confirmed the use of TetCT as a monotherapy for the treatment of lung adenocarcinoma. More extensive prospective clinical studies are needed to determine the safety and efficacy of this agent and to evaluate its potential as a treatment for lung adenocarcinoma.

To our best knowledge, we showed that TetC was a novel powerful anti-tumor drug. Our study first displayed that TetC triggered ferroptosis in LUAD cells by inhibiting the SLC7A11/GPX4 axis. This compound may lay a promising new approach to further LUAD clinical treatment.

## Data Availability

Data will be made available on request.

## References

[1] Sung H, Ferlay J, Siegel RL, Laversanne M, Soerjomataram I (2021). Global cancer statistics 2020: GLOBOCAN estimates of incidence and Mortality worldwide for 36 cancers in 185 countries. CA Cancer J Clin.

[2] Zhang Y, Fu F, Chen H (2020). Management of ground-glass opacities in the lung cancer spectrum. Ann Thorac Surg.

[3] Dixon SJ, Lemberg KM, Lamprecht MR, Skouta R, Zaitsev EM, Gleason CE (2012). Ferroptosis: an iron-dependent form of nonapoptotic cell death. Cell.

[4] Yan HF, Zou T, Tuo QZ, Xu S, Li H, Belaidi AA (2021). Ferroptosis: mechanisms and links with diseases. Signal Transduct Target Ther.

[5] Yang WS, Stockwell BR (2016). Ferroptosis: death by lipid peroxidation. Trends Cell Bio.

[6] Hangauer MJ, Viswanathan VS, Ryan MJ, Bole D, Eaton JK, Matov A (2017). Drug-tolerant persister cancer cells are vulnerable to GPX4 inhibition. Nature.

[7] Lo M, Wang YZ, Gout PW (2008). The x(c)- cystine/glutamate antiporter: a potential target for therapy of cancer and other diseases. J Cell Physiol.

[8] Conrad M, Sato H (2012). The oxidative stress-inducible cystine/glutamate antiporter, system x (c) (-): cystine supplier and beyond. Amino Acids.

[9] Chen X, Li J, Kang R, Klionsky DJ, Tang D (2021). Ferroptosis: machinery and regulation. Autophagy.

[10] Luo Y, Gao X, Zou L, Lei M, Feng J, Hu Z (2021). Bavachin induces ferroptosis through the STAT3/P53/SLC7A11 axis in osteosarcoma cells. Oxid Med Cell Longev.

[11] Yang J, Zhou Y, Xie S, Wang J, Li Z, Chen L (2021). Metformin induces ferroptosis by inhibiting UFMylation of SLC7A11 in breast cancer. J Exp Clin Cancer Res.

[12] Ye Z, Zhuo Q, Hu Q, Xu X, Mengqi L, Zhang Z (2021). FBW7-NRA41-SCD1 axis synchronously regulates apoptosis and ferroptosis in pancreatic cancer cells. Redox Biol.

[13] Luan F, He X, Zeng N (2020). Tetrandrine: a review of its anticancer potentials, clinical settings, pharmacokinetics and drug delivery systems. J Pharm Pharmacol.

[14] Zhou Y, Mu L, Liu XL, Li Q, Ding LX, Chen HC (2019). Tetrandrine inhibits proliferation of colon cancer cells by BMP9/ PTEN/ PI3K/AKT signaling. Genes Dis.

[15] Wang N, Yang S, Tan T, Huang Y, Chen Y, Dong C (2021). Tetrandrine suppresses the growth of human osteosarcoma cells by regulating multiple signaling pathways. Bioengineered.

[16] Sun J, Zhang Y, Zhen Y, Cui J, Hu G, Lin Y (2019). Antitumor activity of tetrandrine citrate in human glioma U87 cells in vitro and in vivo. Oncol Rep.

[17] Chen Z, Zhao L, Zhao F, Yang G, Wang JJ (2018). Tetrandrine suppresses lung cancer growth and induces apoptosis, potentially via the VEGF/HIF-1α/ICAM-1 signaling pathway. Oncol Lett.

[18] Chow LWC, Cheng KS, Leong F, Cheung CW, Shiao LR, Leung YM (2019). Enhancing tetrandrine cytotoxicity in human lung carcinoma A549 cells by suppressing mitochondrial ATP production. Naunyn Schmiedebergs Arch Pharmacol.

[19] Yin JM, Lin YJ, Fang WW, Zhang X, Wei J, Hu G (2022). Tetrandrine citrate suppresses breast cancer via depletion of glutathione peroxidase 4 and activation of nuclear receptor coactivator 4-mediated ferritinophagy. Front Pharmacol.

[20] Lei G, Zhuang L, Gan B (2022). Targeting ferroptosis as a vulnerability in cancer. Nat Rev Cancer.

[21] Battaglia AM, Chirillo R, Aversa I, Sacco A, Costanzo F, Biamonte F (2020). Ferroptosis and cancer: mitochondria meet the “Iron Maiden” cell death. Cells.

[22] Jiang X, Stockwell BR, Conrad M (2021). Ferroptosis: mechanisms, biology and role in disease. Nat Rev Mol Cell Biol.

[23] Mou Y, Wang J, Wu J, He D, Zhang C, Duan C (2019). Ferroptosis, a new form of cell death: opportunities and challenges in cancer. J Hematol Oncol.

[24] Tang X, Ding H, Liang M, Chen X, Yan Y, Wan N (2021). Curcumin induces ferroptosis in non-small-cell lung cancer via activating autophagy. Thorac Cancer.

[25] Zhang Q, Yi H, Yao H, Lu L, He G, Wu M (2021). Artemisinin derivatives inhibit non-small cell lung cancer cells through induction of ROS-dependent Apoptosis/Ferroptosis. J Cancer.

[26] Zhang W, Gong M, Zhang W, Mo J, Zhang S, Zhu Z (2022). Thiostrepton induces ferroptosis in pancreatic cancer cells through STAT3/GPX4 signalling. Cell Death Dis.

[27] Cao JY, Dixon SJ (2016). Mechanisms of ferroptosis. Cell Mol Life Sci.

[28] Zeng X, An H, Yu F, Wang K, Zheng L, Zhou W (2021). Benefits of iron chelators in the treatment of Parkinson’s disease. Neurochem Res.

[29] Zhou Y, Zhou H, Hua L, Hou C, Jia Q, Chen J (2021). Verification of ferroptosis and pyroptosis and identification of PTGS2 as the hub gene in human coronary artery atherosclerosis. Free Radic Biol Med.

[30] Tian Y, Lu J, Hao X, Li H, Zhang G, Liu X (2020). FTH1 inhibits ferroptosis through ferritinophagy in the 6-OHDA model of Parkinson’s disease. Neurotherapeutics.

[31] Antonio Ayala A, Muñoz MF, Argüelles S (2014). Lipid peroxidation: production, metabolism, and signaling mechanisms of malondialdehyde and 4-hydroxy-2- nonenal. Oxid Med Cell Longev.

[32] Tang D, Chen X, Kang R, Kroemer G (2021). Ferroptosis: molecular mechanisms and health implications. Cell Res.

[33] Koppula P, Zhuang L, Gan B (2021). Cystine transporter SLC7A11/xCT in cancer: ferroptosis, nutrient dependency, and cancer therapy. Protein Cell.

[34] Lang X, Green MD, Wang W, Yu J, Choi JE, Jiang L (2019). Radiotherapy and immunotherapy promote tumoral lipid oxidation and ferroptosis via synergistic repression of SLC7A11. Cancer Discov.

[35] Ursini F, Maiorino M (2020). Lipid peroxidation and ferroptosis: the role of GSH and GPx4. Free Radic Biol Med.

[36] Li J, Cao F, Yin HL, Huang ZJ, Lin ZT, Mao N (2020). Ferroptosis: past, present and future. Cell Death Dis.

[37] Yuan B, Liao F, Shi ZZ, Ren Y, Deng XL, Yang TT (2020). Dihydroartemisinin inhibits the proliferation, colony formation and induces ferroptosis of Lung Cancer cells by inhibiting PRIM2/SLC7A11 Axis. Onco Targets Ther.

[38] Wang Z, Zhang X, Tian X, Yang Y, Ma L, Wang J (2021). CREB stimulates GPX4 transcription to inhibit ferroptosis in lung adenocarcinoma. Oncol Rep.

